# Molecular genetic contributions to socioeconomic status and intelligence

**DOI:** 10.1016/j.intell.2014.02.006

**Published:** 2014-05

**Authors:** Riccardo E. Marioni, Gail Davies, Caroline Hayward, Dave Liewald, Shona M. Kerr, Archie Campbell, Michelle Luciano, Blair H. Smith, Sandosh Padmanabhan, Lynne J. Hocking, Nicholas D. Hastie, Alan F. Wright, David J. Porteous, Peter M. Visscher, Ian J. Deary

**Affiliations:** a Centre for Cognitive Ageing and Cognitive Epidemiology, University of Edinburgh, 7 George Square, Edinburgh EH8 9JZ, UK; b Department of Psychology, University of Edinburgh, Edinburgh EH8 9JZ, UK; c Medical Genetics Section, Centre for Genomic and Experimental Medicine, Institute of Genetics and Molecular Medicine, University of Edinburgh, Edinburgh EH4 2XU, UK; d Medical Research Council Human Genetics Unit, Institute of Genetics and Molecular Medicine, University of Edinburgh, Edinburgh EH4 2XU, UK; e Medical Research Institute, University of Dundee, Dundee DD2 4RB, UK; f British Heart Foundation Glasgow Cardiovascular Research Centre, Institute of Cardiovascular and Medical Sciences, College of Medical, Veterinary and Life Sciences, University of Glasgow, Glasgow G12 8TA, UK; g Musculoskeletal Research Programme, Division of Applied Medicine, University of Aberdeen, Aberdeen AB25 2ZD, UK; h University of Queensland Diamantina Institute, The University of Queensland, Princess Alexandra Hospital, Brisbane 4072, QLD, Australia; i Queensland Brain Institute, The University of Queensland, Brisbane 4072, QLD, Australia

**Keywords:** Generation Scotland, Intelligence, Education, Socioeconomic status, Genetics

## Abstract

Education, socioeconomic status, and intelligence are commonly used as predictors of health outcomes, social environment, and mortality. Education and socioeconomic status are typically viewed as environmental variables although both correlate with intelligence, which has a substantial genetic basis. Using data from 6815 unrelated subjects from the Generation Scotland study, we examined the genetic contributions to these variables and their genetic correlations. Subjects underwent genome-wide testing for common single nucleotide polymorphisms (SNPs). DNA-derived heritability estimates and genetic correlations were calculated using the ‘Genome-wide Complex Trait Analyses’ (GCTA) procedures. 21% of the variation in education, 18% of the variation in socioeconomic status, and 29% of the variation in general cognitive ability was explained by variation in common SNPs (SEs ~ 5%). The SNP-based genetic correlations of education and socioeconomic status with general intelligence were 0.95 (SE 0.13) and 0.26 (0.16), respectively. There are genetic contributions to intelligence and education with near-complete overlap between common additive SNP effects on these traits (genetic correlation ~ 1). Genetic influences on socioeconomic status are also associated with the genetic foundations of intelligence. The results are also compatible with substantial environmental contributions to socioeconomic status.

## Highlights

• Generation Scotland is a large family-based cohort of ~ 24,000 people.
• We investigate the genetic influences on education, SES, and intelligence.
• Both DNA-based (subset of ~ 6500) and pedigree-based analyses are used.
• Genetic effects on SES and education are linked to the genetic basis of intelligence.
• There are also substantial environmental effects on all three traits.

## 1 Introduction

Three important, inter-correlated variables that psycho-social scientists bring to bear on social and health-related topics are: intelligence, education, and socioeconomic status. The causes of the associations between these three variables are poorly understood (Deary & Johnson, 2010). All three are used as predictors of people's health outcomes, social environment, and mortality (Batty, Kivimaki, & Deary, 2010; Calvin, Batty, Lowe, & Deary, 2011; Calvin, Deary, et al., 2011; Deary & Batty, 2007; Marmot et al., 1991; Richards et al., 2009). Therefore, disentangling their interplay is important in human life-course research (Deary & Johnson, 2010; Richards & Sacker, 2003; Singh-Manoux, Richards, & Marmot, 2005).

To better understand how intelligence, education, and socioeconomic status are related, we can examine the genetic foundations of these variables and their associations. Twin studies indicate that intelligence has a high heritability in adulthood (Deary, Johnson, & Houlihan, 2009) (around 70%) and that there is a strong genetic correlation between intelligence and education (Calvin et al., 2012). However, twin studies have long been subjected to criticisms on methodological grounds (Plomin, 2012; Trzaskowski et al., 2013) and researchers are now turning to analyses based on DNA differences to obtain heritability estimates of such complex traits. For example, when considered simultaneously, variants in linkage disequilibrium with common single nucleotide polymorphisms (SNPs) explain around 40–50% of the variation in cognitive ability in older people (Davies et al., 2011).

Although educational attainment is often treated as an environmental factor (Deary & Johnson, 2010), common genetic variants explain around 15% of this variation (highest degree completed) (Benjamin et al., 2013) and 22.4% of the variation in years of education (Rietveld et al., 2013). A GWAS meta-analysis of education (n = 126,559) yielded three replicated SNP associations (Rietveld et al., 2013). In addition, a polygenic prediction function, derived from the SNPs most predictive of education, explained 2.5% of the variance in cognitive function (Rietveld et al., 2013). These results suggest that there are genetic influences on people's differences in educational attainment, and it is possible that this is in part explained by education's correlation with the behavioural trait of measured intelligence.

More so than education, socioeconomic status is viewed as an environmental variable. However, one may ask whether variation in the different measures that are used to describe people's socioeconomic status have some genetic contributions.

Apart from socioeconomic status's role as a main effect (or exposure, in epidemiological parlance) that could be partially genetic in origin, it also has a potentially important role as a moderator of genetic influences on intelligence. Socioeconomic status has been shown to interact with intelligence test scores to yield different heritability estimates of intelligence within different social strata. Such gene–environment interactions were described by Turkheimer, Haley, Waldron, D'Onofrio, and Gottesman (2003), who found a greater genetic influence on differences in intelligence in groups from a more affluent background. By contrast, in the lowest socioeconomic group, differences in cognitive ability were almost entirely environmental in origin. However, this effect has not always been replicated (Hanscombe et al., 2012).

Here, we perform univariate and bivariate genetic analyses for education, socioeconomic status, and intelligence. Previous univariate and bivariate heritability studies of these variables have mostly been based on twin and family studies. These models assume random mating of parents and identical environmental exposure for the twins. Such assumptions have been questioned, and the samples used in such analyses are not necessarily population-representative. We do not wish to suggest that the conclusions based on these samples and models are incorrect, but there is now a new and independent way to study genetic contributions to, and genetic correlations between, complex traits that requires neither these assumptions nor such non-representative samples. Molecular (SNP) data derived from testing DNA samples can now be used in unrelated, population-based samples to estimate the degree to which phenotypic similarities can be explained by genetic similarities. The present study's sample, Generation Scotland: the Scottish Family Health Study (Smith et al., 2006, 2012), is a large (n ~ 24,000), family-structured, population-based cohort that allows us to examine both the molecular and the pedigree-based approaches.

## 2 Methods

Generation Scotland: the Scottish Family Health Study (Smith et al., 2006, 2012) is a family-structured, population-based cohort study recruited between 2006 and 2011. Regional sampling occurred in Glasgow, Tayside, Ayrshire, Arran, and North-East Scotland. Participants were recruited through general medical practitioners (GPs) (95% of probands) and via word-of-mouth or direct publicity. We refer to the first member of any one family to be recruited into GS:SFHS as the proband. Probands (n = 7953) were aged between 35 and 65 years and were registered to GP surgeries that were willing to participate in the study. Probands' family members were also recruited, yielding a total sample size of 24,084 with an age range between 18 and 100 and up to three generations per family. There were 5628 participating families with a mean number of 4.03 members per family and a maximum number of 37. There were 1395 singletons. A full description of the cohort is provided elsewhere (Smith et al., 2006, 2012) and online at http://www.generationscotland.org/.

### 2.1 Ethics statement

All components of GS:SFHS received ethical approval from the NHS Tayside Committee on Medical Research Ethics (REC Reference Number: 05/S1401/89). GS:SFHS has been granted Research Tissue Bank status by the Tayside Committee on Medical Research Ethics (REC Reference Number: 10/S1402/20), providing generic ethical approval for a wide range of uses within medical research.

### 2.2 Genotyping sample

Selection criteria for genome-wide analysis of 10,000 participants were: Caucasian ethnicity, born in the UK (prioritising those born in Scotland), and full phenotype data available from attendance at a Generation Scotland research clinic. The participants were also selected to have chosen to consent to be re-contacted for new research and all can be linked to National Health Service medical records using the Community Health Index (CHI) number. The selected samples include 446 DNAs that have also been exome sequenced primarily for analyses of depression (150 extreme [early onset] recurrent major depressive disorder [rMDD]), cognition (150 samples from participants with high general cognitive ability [denoted g]) and 146 family representatives of sequenced participants with MDD, comprising parent–child trios and quads. A further 3234 DNAs from trios and quads, 807 participants with measured rMDD, and 5513 unrelated participants make up the 10,000 sample total.

### 2.3 DNA genotyping

Blood samples (or saliva from postal and a few clinical participants) from GS:SFHS participants were collected, processed and stored using standard operating procedures and managed through a laboratory information management system at the Wellcome Trust Clinical Research Facility Genetics Core, Edinburgh (Kerr et al., 2013). The yield of DNA was measured using PicoGreen and normalised to 50 ng/μl before genotyping. Genotyping was performed using the Illumina HumanOmniExpressExome-8 v1.0 DNA Analysis BeadChip and Infinium chemistry (Gunderson, 2009). In summary, this consists of three steps: (i) whole genome amplification, (ii) fragmentation followed by hybridisation, and (iii) single-base extension and staining. For each of the samples, 4 μl of DNA normalised to 50 ng/μl was used. The Arrays were imaged on an Illumina HiScan platform and genotypes were called automatically using GenomeStudio Analysis software v2011.1. After quality control, there were a total of 594,824 SNPs available for analysis on 9863 individuals.

To remove a shared environment influencing the associations, a genetic cut-off of 0.025 was specified, which excludes individuals who are more closely related than second cousins (Yang, Lee, Goddard, & Visscher, 2011; Yang et al., 2010). Rare SNPs with a minor allele frequency less than 1% were removed prior to the analyses. This left an unrelated, genotyped analysis sample of 6815 individuals.

### 2.4 Cognition, education, and socioeconomic status

Four cognitive tests were administered to GS:SFHS participants. This included measures of processing speed (Wechsler Digit Symbol Substitution Task — DST, (Wechsler, 1998a)), verbal declarative memory (Wechsler Logical Memory Test — LM, (Wechsler, 1998b)), and executive function (Verbal Fluency Test — VFT, (Lezak, 1995)) and vocabulary/crystallised-type intelligence (Mill Hill Vocabulary Scale — MHVS, (Raven, Court, & Raven, 1977)). Principal component analysis was applied to the scores from these four tests to obtain a general intelligence component score, which was named g. The score was based on the first unrotated principal component, which explained 42% of the variance in the complete sample and 45% of the variance in the unrelated, genotyped sample. The individual tests had strong loadings in both groups (complete sample: DST = 0.59, LM = 0.63, VFT = 0.72, MHVS = 0.66; unrelated, genotyped sample: DST = 0.65, LM = 0.64, VFT = 0.72, MHVS = 0.69). A similar procedure was used to obtain a fluid-type general intelligence measure, g_f_, which was based on the scores from the DST, LM, and VFT.

Education was measured as years of full time, formal education, on an ordinal scale from 0 to 10.

The Scottish Index of Multiple Deprivation 2009 (SIMD, http://www.scotland.gov.uk/topics/statistics/simd/) was used to assess socioeconomic status. Briefly, small areas in Scotland are ranked according to seven domains: income, employment, health, education, geographic access, crime, and housing. The range of ranks goes from the most deprived (rank 1) to the least deprived (rank 6505). A rank-based inverse normal transformation was applied to SIMD scores, pictured in Supplementary Fig. 1, prior to the analyses.

The Scottish Index of Multiple Deprivation 2009 (SIMD, http://www.scotland.gov.uk/topics/statistics/simd/) was used to assess socioeconomic status. Briefly, small areas in Scotland are ranked according to seven domains: income, employment, health, education, geographic access, crime, and housing. The range of ranks goes from the most deprived (rank 1) to the least deprived (rank 6505). A rank-based inverse normal transformation was applied to SIMD scores, pictured in Supplementary Fig. 1, prior to the analyses.

### 2.5 Statistical analyses

Prior to the main analyses, the traits (education, g, and SIMD) were adjusted for age, sex, and, in the molecular analyses, population stratification (the first six principal components) using linear regression. Residuals from these models were used as the dependent variables of interest. The number of ancestry components was determined by comparing the log-Likelihoods and residual errors (square root of the residual variance) from linear regression models of the three traits on age, sex, and up to twenty principal components (Supplementary Fig. 2). Based on these data, the optimal number of components to adjust for was six.

Prior to the main analyses, the traits (education, g, and SIMD) were adjusted for age, sex, and, in the molecular analyses, population stratification (the first six principal components) using linear regression. Residuals from these models were used as the dependent variables of interest. The number of ancestry components was determined by comparing the log-Likelihoods and residual errors (square root of the residual variance) from linear regression models of the three traits on age, sex, and up to twenty principal components (Supplementary Fig. 2). Based on these data, the optimal number of components to adjust for was six.

Initially, univariate and bivariate heritability analyses were run on the three traits using pedigree information to define relatedness. This approach does not require genetic information; therefore, the full sample of Generation Scotland participants was analysed (n_range_ = 23,673), giving familiality estimates for the traits. Briefly, genetic and environmental variances are partitioned using a linear mixed model that compares phenotypic covariance between pairs of relations. Relatedness is defined theoretically: e.g., the genetic correlation between parent and offspring is 0.5 and 0.25 for grandparent and grandchild. To account for siblings sharing environmental variance, a maternal random effect was considered. This improved the model fit in all analyses with the exception of those (univariate and bivariate) involving SIMD. However, even in the models where the fit was improved, the effect size for the maternal component was small. We did not model the potential shared environmental effects in the extended pedigrees. The models fitted here were run in ASReml-R (Butler, Cullis, Gilmour, & Gogel, 2007; R Core Team, 2013) and are analogous to those previously reported by Luciano et al. (2010) based on a subsample of GS:SFHS participants.

In the molecular approach, univariate genome-wide complex trait analyses (Yang et al., 2010, 2011) (GCTA) were run on the three traits to determine the proportion of variance explained by genetic variants in linkage disequilibrium with common SNPs. GCTA fits a standard linear mixed model but includes SNPs as random effects in order to investigate how genetic similarities predict phenotypic similarities. To test the hypothesis proposed by Turkheimer et al. (2003)—that heritability might be lower in people from more deprived social backgrounds—the univariate model for g was re-run, splitting by median SIMD. Bivariate GCTA (Lee, Yang, Goddard, Visscher, & Wray, 2012) was then run to investigate the SNP-based genetic correlations between education, g, and SIMD. To account for the over-sampling of individuals with depression in the GWAS cohort, all GCTA analyses were re-run after excluding this sub-group along with those selected for having very high cognitive scores (n_analysis_ = 5991).

## 3 Results

Genome-wide SNP data were available on 6815 unrelated Generation Scotland participants; median age 57 years (IQR 49–63), and 4002 (59%) were female (Table 1). The majority of the subjects (53%) had between 10 and 13 years of education. Around 17% of the sample came from the most deprived 30% of SIMD datazones (ranked between 1 and 1952). There were no notable differences between the genotyped subgroup and the full sample, with the exception of age. The full sample (n ~ 23,673) was younger (median age 49 years, IQR 36–59). Age- and sex-adjusted phenotypic correlations in the genotyped sample (Table 2) showed an association between g and education (Pearson r ~ 0.38). SIMD correlated between 0.21 and 0.25 with both cognition and education. Correlations between pairs of relatives are presented in Supplementary Table 1.

The family-based pedigree analyses (Table 3, Supplementary Tables 2 and 3) yielded univariate estimates of narrow-sense heritability that were 0.41 (SE 0.02) for education, 0.54 (SE 0.02) for g, and 0.71 (SE 0.01) for SIMD. The bivariate analyses found that the genetic correlations were 0.40 (SE 0.02) between SIMD and g, 0.48 (SE 0.02) between SIMD and education, and 0.65 (SE 0.02) between education and g. The environmental correlations were small (range − 0.18 to 0.12). Bivariate heritability estimates were high for all pairs of traits, ranging between 0.78 and 0.88.

In the molecular approach, univariate GCTA analyses found the proportion of variance in g explained by common SNPs to be 0.29 (SE 0.05) (Table 3, Supplementary Table 4). The corresponding figures for the individual tests that were used to derive g ranged between 0.13 for Logical Memory and 0.39 for Mill Hill Vocabulary (Supplementary Table 4). There was a modest difference in the proportion of variance in g explained by the SNPs upon separating the cohort by median SIMD (Supplementary Table 4). The proportion of variance in g explained by common SNPs for those with a SIMD rank above the median was 0.31 compared to 0.15 for those below the median. The standard errors for these quantities were relatively large (both 0.11).

The variance in education and SIMD explained by common SNPs was similar: 0.21 (SE 0.05) and 0.18 (SE 0.05), respectively (Table 3, Supplementary Table 4). After partialling out the effect of fluid-type intelligence (g_f_) or crystallised-type intelligence (MHVS), the education estimates decreased to 0.15 (SE 0.06) and 0.08 (SE 0.05). There were smaller differences in the corresponding partialled effect sizes for SIMD: estimates were 0.15 (SE 0.06) and 0.13 (SE 0.06), respectively.

In the bivariate GCTA models, the genetic correlation between g and education was 0.95 (SE 0.13) (Table 3, and Supplementary Table 5). This suggests that the genetic influences on the two traits overlap substantially. The corresponding association between g and SIMD was moderate: 0.26 (SE 0.16). The genetic correlation between the two ‘environmental’ variables, education and SIMD, was 0.45 (SE 0.18). A principal component analysis on the bivariate genetic correlation matrix (including g, education, and SIMD) yielded a first unrotated principal component that explained 72% of the variance. Table 3 also provides the bivariate environmental correlations. These show a modest (16–24%) overlap in the non-shared environmental influences on the traits. Bivariate heritability estimates, which represent the proportion of the phenotypic correlation that can be explained by additive SNP effects, were moderate-to-large: 24% for g and SIMD, 41% for education and SIMD, and 59% for g and education.

To account for potential domain-specific variability, sensitivity analyses were performed for g split into general fluid-type intelligence (g_f_; based on three tests), and crystallised-type intelligence (a single test — MHVS) components (Supplementary Table 5). The genetic correlations between education and both g_f_ and MHVS were large, 0.83 (SE 0.17) and 0.92 (0.11), respectively. The genetic correlation between SIMD and g_f_ was small: 0.18 (SE 0.19). The corresponding figure for SIMD and MHVS was larger and significantly different from zero: 0.42 (SE 0.14). Further sensitivity analyses that accounted for the over-sampling of individuals with depression had no effect on the results (Supplementary Tables 6 and 7).

## 4 Discussion

There are some key findings from these analyses. Firstly, the proportion of variance in education explained by common SNPs is 21%. Education had an almost-perfect genetic correlation with g, and 59% of their phenotypic correlation can be explained variation in common SNPs. Secondly, we observed a genetic contribution to variation in socioeconomic status (18% explained by common SNPs), 26% of which was shared with genes that influence intelligence. Around 24% of their phenotypic correlation can be explained by common SNPs. This is important for those studying inequalities in health, because cognitive ability and socioeconomic status are correlated predictors of health outcomes. Thirdly, we found a slight difference between the proportion of variance in g explained by SNPs for those above or below the median socioeconomic level (31% versus 15%). This is in the same direction as reported by Turkheimer et al., 2003, although there were large standard errors about both of our estimates (11%), which means that the difference between them is not statistically significant.

We note that, because we examined only a defined, albeit large set of common SNPs and not other types of genetic variation, the univariate estimates of heritability provided here by the GCTA analyses are lower-bound estimates of narrow sense heritability, such as are contributed to by genetic variants in linkage disequilibrium with those common SNPs that were typed. However, assuming that the genetic correlation is the same for tagged and untagged causal variants, then the GCTA-derived estimate of this quantity will be unbiased (Trzaskowski et al., 2013).

Comparing the univariate GCTA results for general intelligence, g, with those previously published show it to be within the range of childhood estimates (Benyamin et al., 2014). The estimates for fluid-type general intelligence, g_f_, are lower than those obtained in a sample of mid-to-late-life adults (Davies et al., 2011). Possible explanations for differences include the relatively large standard errors from previous analyses, the wide spread of ages assessed in Generation Scotland, phenotypic heterogeneity (the g_f_ component has been based on different tests in different studies), and the small (only three) set of cognitive tests used to form g_f_ in the present study. By contrast the vocabulary-based estimate of crystallised intelligence had a higher proportion (42%) of variance explained by common SNPs. This figure is similar to those reported previously (Davies et al., 2011).

Our univariate GCTA estimate for education provides a replication of previous findings (Benjamin et al., 2013; Rietveld et al., 2013). Moreover, our corresponding estimate for socioeconomic status closely matches the figure reported by Trzaskowski et al. (2014). In their molecular study of family socioeconomic status in children, they that found 19% (SE 12%) of the variance was explained by common SNPs at age 2 (n = 2864), with 20% (SE 12%) of the variance explained at age 7 (n = 2679). The present study provides similar estimates, but with considerably smaller standard errors.

The genetic correlations from the bivariate GCTA indicated notable pairwise associations among the traits. In particular, there was a close to a complete overlap between the additive genetic effects that influence education and g. The strength of the relationship can also be seen in the univariate GCTA analysis for education that had additional adjustments for g_f_ or MHVS. The genetic correlations between SIMD and the other traits were of a moderate size, indicating a significant overlap with additive genetic influences on intelligence (particularly MHVS; Supplementary Table 5) and education. Moreover, the bivariate heritability estimates suggest that common SNPs explain 24–59% of the phenotypic correlations. Bivariate GCTA estimates between childhood intelligence (at ages 7 and 12) and family-based socioeconomic status (at age 7) have been presented in the study of Trzaskowski et al. (2014). Using samples of 1750 subjects at age 7, and 2013 subjects at age 12, they found genetic correlations with age 7 family socioeconomic status of 1.00 (SE 0.47) and 0.66 (0.31), respectively. Despite the relatively small sample size and resulting large standard errors for these estimates, they are still significantly different from zero.

In conclusion, using DNA SNP data we found modest genetic contributions to differences in socioeconomic status that have measurable association with the genetic basis of intelligence differences and education. The implication from these analyses is that socioeconomic status and education have, in part, genetic causes that are shared with the genetic contributions to measured intelligence. In a discussion article on the causes of the association between intelligence and education (Deary & Johnson, 2010) we noted that some researchers treated education as if it were an almost-entirely environmental variable—education was something that happened to people and changed them—whereas other researchers treated education as a proxy for mostly genetically-influenced prior cognitive ability. We appealed for more nuanced thinking, and for arguments to be based more on data and less on preconceptions. The present study's results add to that effort. It is useful to know that measures used to indicate ‘environmental’ qualities largely appear to do that. The results are therefore compatible with substantial—but not exclusive—environmental contributions to socioeconomic status and education.

The following are the supplementary data related to this article.

Supplementary Fig. 1 Histogram of Scottish Index of Multiple Deprivation (SIMD) ranks from 2009 in the full Generation Scotland cohort of 23,673 and the unrelated, genotyped subset of 6815 subjects.

Supplementary Fig. 2 Comparison of log-likelihoods and residual errors for linear regression models of age-, sex-, and population stratification (up to 20 principal components)-adjusted cognition, education, and SIMD.

Supplementary Table 1: Pearson correlations of general intelligence, g, between pairs of relatives.
Supplementary Table 2: Age- and sex-adjusted univariate pedigree models for cognition, education, and social class with and without maternal effects.
Supplementary Table 3: Age- and sex-adjusted bivariate pedigree models for cognition, education, and social class.
Supplementary Table 4: Age-, sex-, and population stratification-adjusted univariate GCTA models for cognition, education, and social class.
Supplementary Table 5: Age-, sex-, and population stratification-adjusted bivariate GCTA models for cognition, education, and social class.
Supplementary Table 6: Age- and sex- and population stratification-adjusted univariate GCTA models for cognition, education, and social class excluding those with depression.
Supplementary Table 7: Age-, sex- and population stratification-adjusted bivariate GCTA models for cognition, education, and social class excluding those with depression.

Supplementary data to this article can be found online at http://dx.doi.org/10.1016/j.intell.2014.02.006.

## Figures and Tables

**Table 1:** Descriptive data for the (unrelated) genotyped and complete Generation Scotland cohorts.

(Unrelated) Genotyped Generation Scotland cohort				Complete Generation Scotland cohort			
Variable	n	Mean (SD), N (%), or median (IQR)		Variable	n	Mean (SD), N (%), or median (IQR)	
*Demographics*				*Demographics*			
Age	6,815	57	49–63	Age	23,673	49	36–59
Sex — Female	6,815	4,002	59	Sex — Female	23,673	13,904	59

*Environmental*				*Environmental*			
Education (years)	6,578			Education (years)	22,406		
0		3	0.04	0		11	0.04
1–4		26	0.40	1–4		67	0.30
5–9		223	3.39	5–9		702	3.13
10–11		2,137	32.49	10–11		5,945	26.53
12–13		1,339	20.36	12–13		4,760	21.24
14–15		901	13.70	14–15		3,196	14.26
16–17		1,128	17.15	16–17		4,568	20.39
18–19		568	8.63	18–19		2,264	10.10
20–21		177	2.69	20–21		619	2.76
22–23		49	0.74	22–23		183	0.82
24 +		27	0.41	24 +		91	0.41
SIMD	6,533	4,548	2,739–5,548	SIMD	20,785	4,331	2,373–5,461

*Cognitive*				*Cognitive*			
DST	6,718	68.4	16.8	DST	20,908	72.2	17.2
VFT	6,736	41.0	12.2	VFT	20,895	39.7	11.7
LM	6,731	30.3	7.9	LM	20,881	31.0	8.0
MHVS	6,694	31.2	4.7	MHVS	20,770	30.1	4.8

SIMD: Scottish Index of Multiple Deprivation, DST: Digit Symbol Test, VFT: Verbal Fluency Test, LM: Logical Memory, MHVS: Mill Hill Vocabulary Scale.

**Table 2:** Age-, and sex-adjusted phenotypic Pearson correlations (SE).

	g	Education	SIMD
g	1	0.38 (0.01)	0.23 (0.01)
Education	0.39 (0.01)	1	0.21 (0.01)
SIMD	0.25 (0.01)	0.22 (0.01)	1

g: general intelligence derived from principal components analysis, SIMD: Scottish Index of Multiple Deprivation. The correlations for the total population are shown on the upper diagonal; the lower diagonal shows the correlations for the unrelated, genotyped sub-sample.

**Table 3:** Univariate and bivariate pedigree and GCTA estimates of heritability.

Pedigree-based estimates^a^					
*Univariate estimates*	n	h^2^	SE		
g	20,522	0.54	0.02		
Education	22,406	0.41	0.02		
SIMD	20,785	0.71	0.01		

*Bivariate estimates*	n	r_G_	SE	r_E_	biv h^2^
g: Education	20,522:22,406	0.65	0.02	0.12	0.78
g SIMD	20,522:20,785	0.40	0.02	− 0.09	0.88
Education : SIMD	22,406:20,785	0.48	0.02	− 0.18	0.79

SNP-based (GCTA) estimatesb, c					

*Univariate estimates*	n	h^2^	SE		
g	6,609	0.29	0.05		
Education	6,578	0.21	0.05		
SIMD	6,533	0.18	0.05		

*Bivariate estimates*	n	r_G_	SE	r_E_	biv h^2^
g: Education	6,609:6,578	0.95	0.13	0.21	0.59
g: SIMD	6,609:6,533	0.26	0.16	0.24	0.24
Education : SIMD	6,578:6,533	0.45	0.18	0.16	0.41

g: general intelligence derived from principal components analysis, SIMD: Scottish Index of Multiple Deprivation, h^2^: narrow-sense heritability, r_G_: genetic correlation, r_E_: environmental correlation, biv h^2^: bivariate heritability.

a Age- and sex-adjusted.

b Age-, sex-, and population stratification-adjusted.

c The univariate estimates give the proportion of variance in the phenotype explained by common genetic variants.
