# Supplementary figures and images for: Molecular genetic contributions to socioeconomic status and intelligence
Source: Intelligence. 2014 May;44(100):26–32. doi: 10.1016/j.intell.2014.02.006 (PMC4051988; doi:10.1016/j.intell.2014.02.006)

**Full Cohort (n=23,673)**

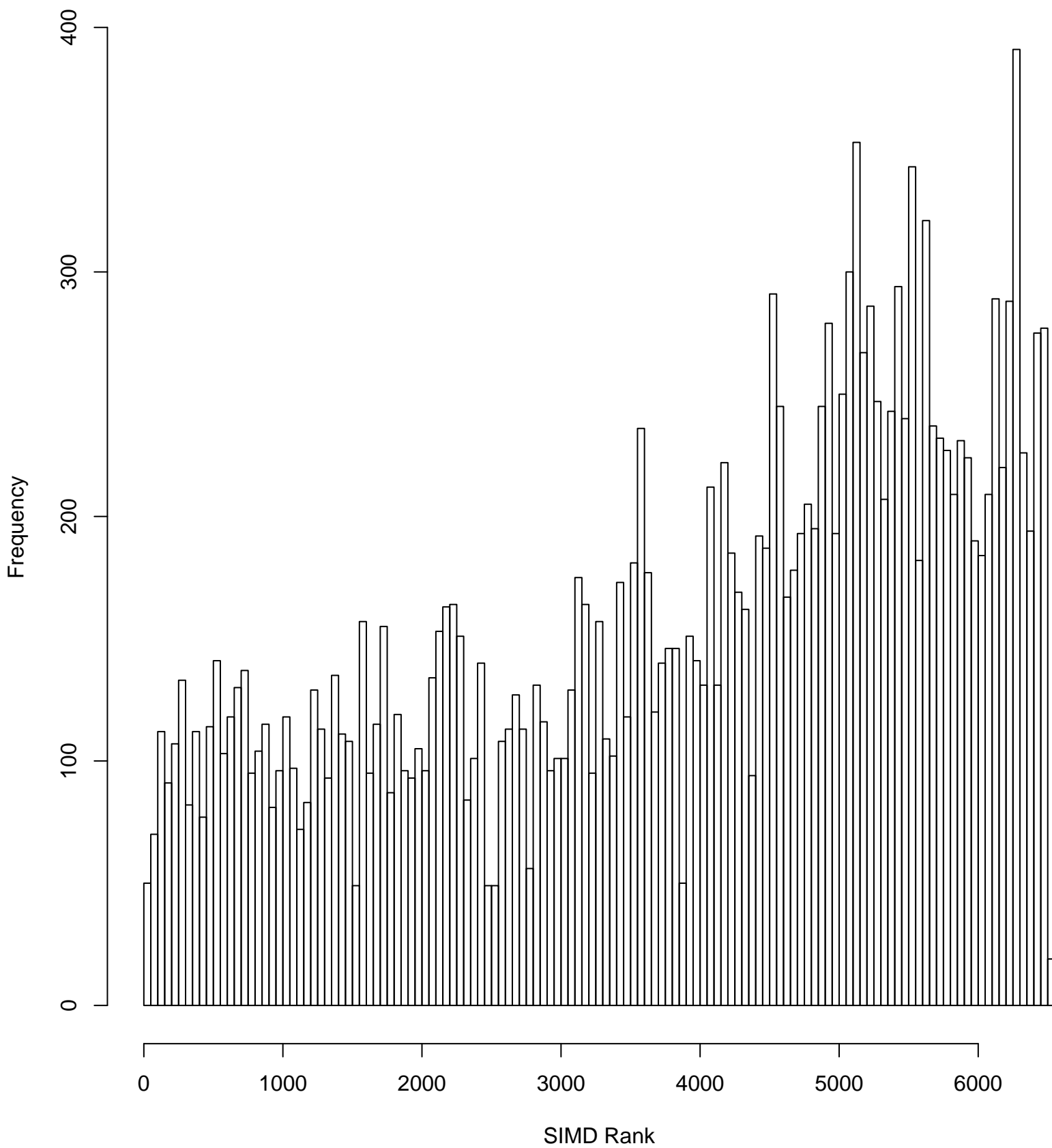

**Unrelated, Gentoyped Cohort (n=6,815)**

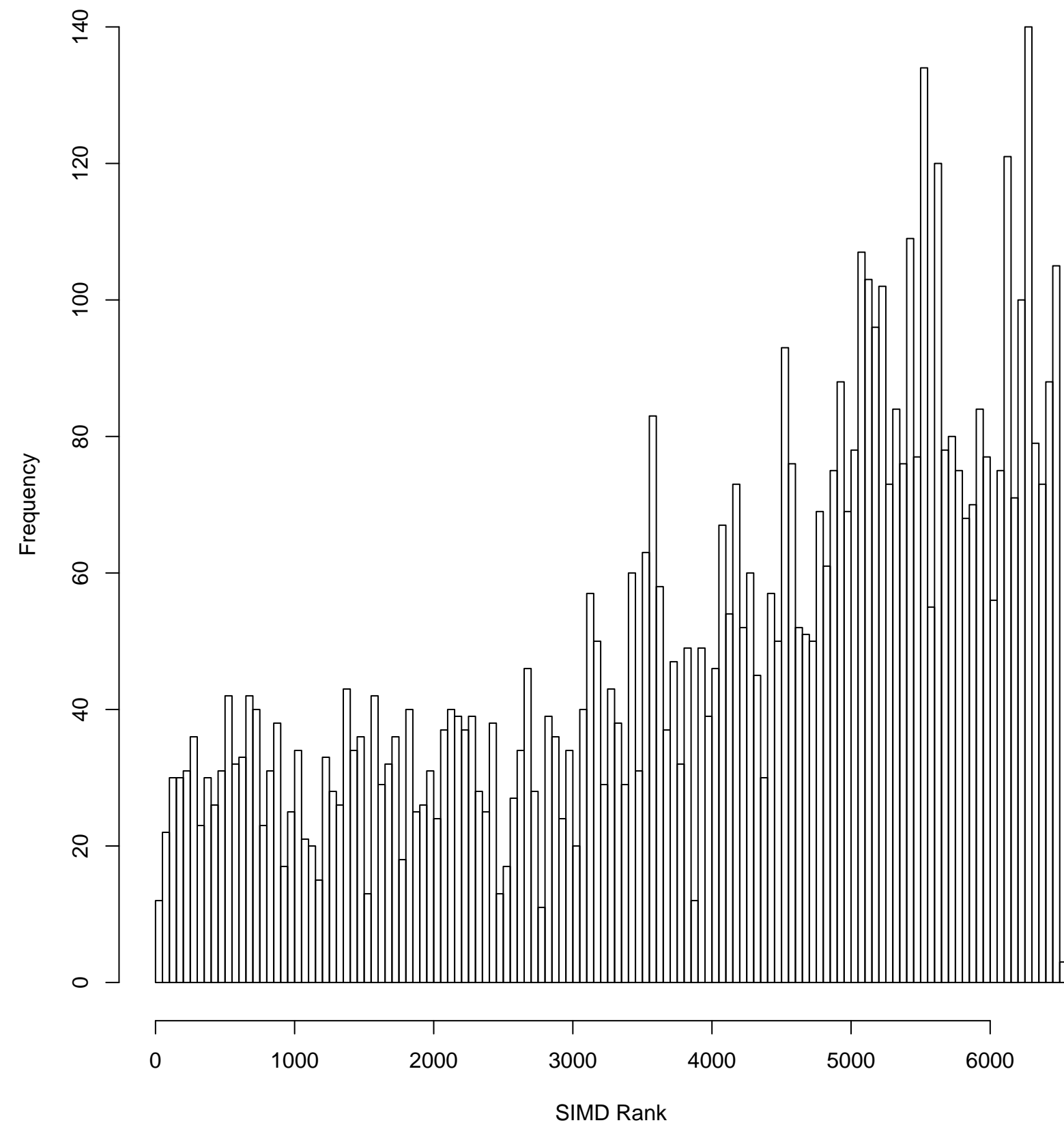

Supplement: Supplementary Fig. 1 — Histogram of Scottish Index of Multiple Deprivation (SIMD) ranks from 2009 in the full Generation Scotland cohort of 23,673 and the unrelated, genotyped subset of 6815 subjects. [file mmc1.pdf]

**g**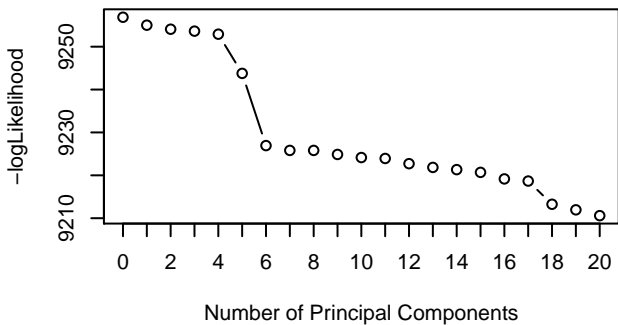**g**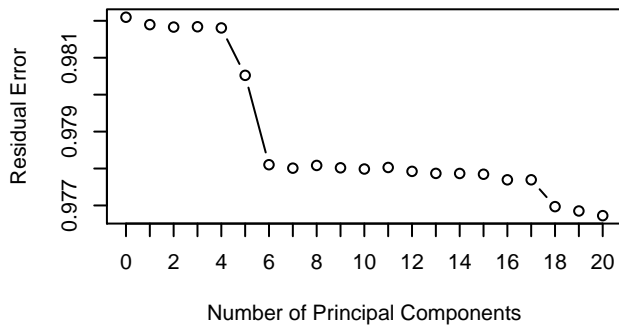**Education**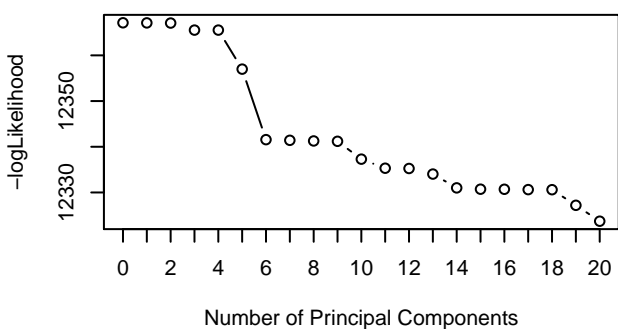**Education**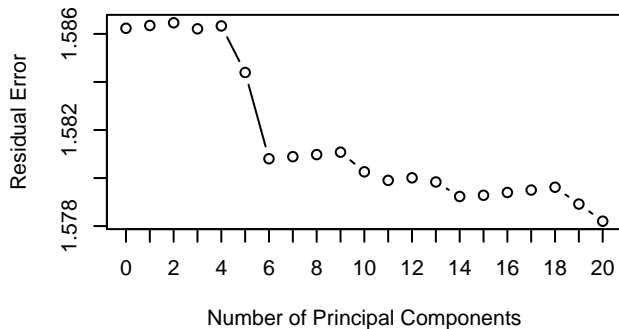**SIMD**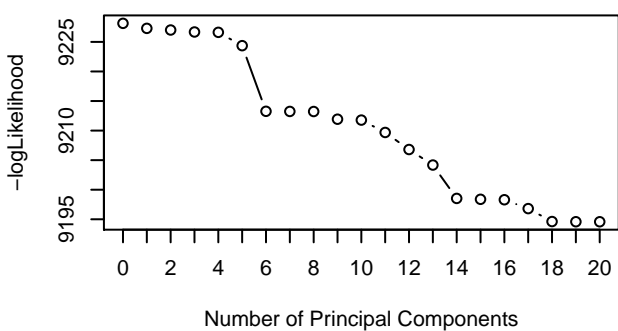**SIMD**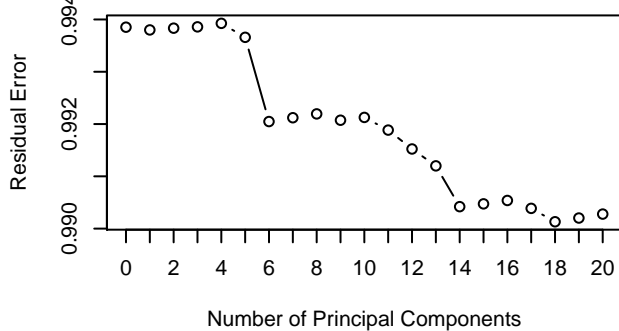

Supplement: Supplementary Fig. 2 — Comparison of log-likelihoods and residual errors for linear regression models of age-, sex-, and population stratification (up to 20 principal components)-adjusted cognition, education, and SIMD. [file mmc2.pdf]
