# Supplementary material for: Molecular genetic contributions to socioeconomic status and intelligence
Source: Intelligence. 2014 May;44(100):26–32. doi: 10.1016/j.intell.2014.02.006 (PMC4051988; doi:10.1016/j.intell.2014.02.006)
Supplement: Supplementary file 3 — Supplementary Table 1: Pearson correlations of general intelligence, g, between pairs of relatives. Supplementary Table 2: Age- and sex-adjusted univariate pedigree models for cognition, education, and social class with and without maternal effects. Supplementary Table 3: Age- and sex-adjusted bivariate pedigree models for cognition, education, and social class. Supplementary Table 4: Age-, sex-, and population stratification-adjusted univariate GCTA models for cognition, education, and social class. Supplementary Table 5: Age-, sex-, and population stratification-adjusted bivariate GCTA models for cognition, education, and social class. Supplementary Table 6: Age- and sex- and population stratification-adjusted univariate GCTA models for cognition, education, and social class excluding those with depression. Supplementary Table 7: Age-, sex- and population stratification-adjusted bivariate GCTA models for cognition, education, and social class excluding those with depression. [file mmc3.doc]

Supplementary Table 1: Pearson correlations of general intelligence, g, between pairs of relatives.

| Relationship | n | Pearson r | SE | P |
| --- | --- | --- | --- | --- |
| First Cousin | 2,430 | 0.15 | 0.02 | <0.001 |
| Avuncular | 6,900 | 0.16 | 0.01 | <0.001 |
| Grandparent-Grandchild | 801 | 0.03 | 0.04 | 0.34 |
| Half-cousin | 8 | -0.74 | 0.28 | 0.038 |
| Half-avuncular | 47 | -0.15 | 0.15 | 0.31 |
| Half-sib | 106 | 0.15 | 0.10 | 0.12 |
| Parent-Child | 10,112 | 0.26 | 0.01 | <0.001 |
| Sib | 9,129 | 0.37 | 0.01 | <0.001 |
| Non-blood relative | 9,916 | 0.17 | 0.01 | <0.001 |

Supplementary Table 2: Age- and sex-adjusted univariate pedigree models for cognition, education, and social class with and without maternal effects.

|  | n | V_G_ | SE | V_M_ | SE | V_R_ | SE | h^2^ | SE |
| --- | --- | --- | --- | --- | --- | --- | --- | --- | --- |
| *Individual cognitive tests* |  |  |  |  |  |  |  |  |  |
| MHVS | 20,770 | 11.437 | 0.343 | - | - | 8.373 | 0.266 | 0.577 | 0.014 |
|  |  | 11.005 | 0.371 | 1.088 | 0.220 | 7.913 | 0.271 | 0.550 | 0.017 |
|  |  |  |  |  |  |  |  |  |  |
| *General cognitive ability* |  |  |  |  |  |  |  |  |  |
| g | 20,522 | 0.554 | 0.017 | - | - | 0.414 | 0.013 | 0.572 | 0.015 |
|  |  | 0.533 | 0.019 | 0.056 | 0.011 | 0.390 | 0.014 | 0.544 | 0.017 |
| g_f_ | 20,664 | 0.421 | 0.015 | - | - | 0.451 | 0.013 | 0.483 | 0.015 |
|  |  | 0.401 | 0.017 | 0.042 | 0.010 | 0.437 | 0.013 | 0.456 | 0.018 |
|  |  |  |  |  |  |  |  |  |  |
| *Environmental variables* |  |  |  |  |  |  |  |  |  |
| Education | 22,406 | 1.151 | 0.038 | - | - | 1.259 | 0.032 | 0.478 | 0.014 |
|  |  | 1.002 | 0.043 | 0.297 | 0.028 | 1.178 | 0.033 | 0.405 | 0.017 |
| SIMD | 20,785 | 0.688 | 0.017 | - | - | 0.284 | 0.012 | 0.708 | 0.013 |
|  |  | 0.688 | 0.017 | 1.7x10^-7^ | 6.9x10^-9^ | 0.284 | 0.012 | 0.708 | 0.013 |

MHVS: Mill Hill Vocabulary Scale, g: general intelligence derived from principal components analysis, g_f_: general fluid-type intelligence derived from principal components analysis, SIMD: Scottish Index of Multiple Deprivation. V_G_: random additive genetic effect, V_M_: random maternal effect, V_E_: residual variance, h^2^: narrow sense heritability.

Supplementary Table 3: Age- and sex-adjusted bivariate pedigree models for cognition, education, and social class.

|  | n | V_G1_ | SE | Cov_G1-G2_ | SE | V_G2_ | SE | V_M1_ | SE | Cov_M1-M2_ | SE | V_M2_ | SE | V_R1_ | SE | Cov_R1-R2_ | SE | V_R2_ | SE |
| --- | --- | --- | --- | --- | --- | --- | --- | --- | --- | --- | --- | --- | --- | --- | --- | --- | --- | --- | --- |
| g : Education | 20,522:22,406 | 0.519 | 0.018 | 0.464 | 0.021 | 0.989 | 0.043 | 0.064 | 0.011 | 0.117 | 0.013 | 0.296 | 0.028 | 0.013 | 0.397 | 0.016 | 0.016 | 1.193 | 0.032 |
| g : SIMD | 20,552:20,785 | 0.544 | 0.017 | 0.243 | 0.012 | 0.683 | 0.017 | - | - | - | - | - | - | 0.420 | 0.013 | -0.032 | 0.009 | 0.288 | 0.012 |
| Education : SIMD | 22,406:20,785 | 1.112 | 0.038 | 0.416 | 0.018 | 0.677 | 0.017 | - | - | - | - | - | - | 1.287 | 0.032 | -0.107 | 0.014 | 0.292 | 0.012 |
| g_f_ : Education | 20,664:22,406 | 0.392 | 0.017 | 0.337 | 0.020 | 0.997 | 0.043 | 0.046 | 0.010 | 0.088 | 0.013 | 0.297 | 0.028 | 0.013 | 0.442 | 0.015 | 0.015 | 1.185 | 0.033 |
| g_f_ : SIMD | 20,664:20,785 | 0.415 | 0.015 | 0.201 | 0.012 | 0.686 | 0.017 | - | - | - | - | - | - | 0.455 | 0.013 | -0.021 | 0.009 | 0.286 | 0.012 |
| MHVS : Education | 20,770:22,406 | 10.701 | 0.362 | 2.267 | 0.095 | 0.981 | 0.042 | 1.305 | 0.206 | 0.564 | 0.058 | 0.295 | 0.027 | 0.268 | 8.043 | 0.070 | 0.070 | 1.201 | 0.032 |
| MHVS : SIMD | 20,770:20785 | 11.174 | 0.337 | 1.076 | 0.054 | 0.678 | 0.017 | - | - | - | - | - | - | 8.555 | 0.264 | -0.201 | 0.040 | 0.291 | 0.012 |

g: general intelligence derived from principal components analysis, SIMD: Scottish Index of Multiple Deprivation, g_f_: general fluid-type intelligence derived from principal components analysis, MHVS: Mill Hill Vocabulary Scale. V_G_*_i_*: random additive genetic effect for trait *i*, V_M_*_i_*: random maternal effect for trait *i*, V_R_*_i_*: residual variance for trait *i*, Cov: covariance between random genetic, maternal, or residual variance effects.

Supplementary Table 4: Age-, sex-, and population stratification^a^-adjusted univariate GCTA models for cognition, education, and social class.

|  | n | V(G) | SE | V(e) | SE | Vp | SE | V(G)/Vp | SE |
| --- | --- | --- | --- | --- | --- | --- | --- | --- | --- |
| *Individual cognitive tests* |  |  |  |  |  |  |  |  |  |
| DST | 6,718 | 38.621 | 11.235 | 174.483 | 11.394 | 213.104 | 3.684 | 0.181 | 0.052 |
| VFT | 6,736 | 30.825 | 7.946 | 116.733 | 8.001 | 147.558 | 2.549 | 0.209 | 0.053 |
| LM | 6,731 | 7.806 | 3.187 | 52.372 | 3.265 | 60.179 | 1.038 | 0.130 | 0.053 |
| MHVS | 6,694 | 7.953 | 1.118 | 12.606 | 1.086 | 20.559 | 0.358 | 0.387 | 0.053 |
|  |  |  |  |  |  |  |  |  |  |
| *General cognitive ability* |  |  |  |  |  |  |  |  |  |
| g | 6,609 | 0.280 | 0.052 | 0.675 | 0.051 | 0.955 | 0.017 | 0.293 | 0.054 |
| g_f_ | 6,648 | 0.180 | 0.047 | 0.695 | 0.047 | 0.875 | 0.015 | 0.205 | 0.053 |
|  |  |  |  |  |  |  |  |  |  |
| *Environmental variables* |  |  |  |  |  |  |  |  |  |
| Education | 6,578 | 0.519 | 0.135 | 1.977 | 0.136 | 2.496 | 0.044 | 0.208 | 0.054 |
| Education (g_f_ adjusted) | 6,422 | 0.334 | 0.124 | 1.930 | 0.127 | 2.264 | 0.040 | 0.148 | 0.055 |
| Education (MHVS adjusted) | 6,470 | 0.172 | 0.112 | 1.891 | 0.116 | 2.063 | 0.036 | 0.083 | 0.054 |
| SIMD | 6,533 | 0.177 | 0.054 | 0.806 | 0.054 | 0.983 | 0.017 | 0.180 | 0.054 |
| SIMD (g_f_ adjusted) | 6,373 | 0.139 | 0.052 | 0.797 | 0.053 | 0.937 | 0.017 | 0.149 | 0.056 |
| SIMD (MHVS adjusted) | 6,416 | 0.123 | 0.051 | 0.803 | 0.052 | 0.926 | 0.016 | 0.132 | 0.055 |
|  |  |  |  |  |  |  |  |  |  |
| *'Turkheimer' estimates* |  |  |  |  |  |  |  |  |  |
| g (below median SIMD) | 3,136 | 0.149 | 0.109 | 0.856 | 0.110 | 1.005 | 0.025 | 0.148 | 0.109 |
| g (above median SIMD) | 3,198 | 0.253 | 0.091 | 0.569 | 0.091 | 0.822 | 0.021 | 0.308 | 0.110 |

DST: Digit Symbol Test, VFT: Verbal Fluency Test, LM: Logical Memory, MHVS: Mill Hill Vocabulary Scale, g: general intelligence derived from principal components analysis, g_f_: general fluid-type intelligence derived from principal components analysis, SIMD: Scottish Index of Multiple Deprivation. V(G): genetic variance, V(e): residual variance, Vp: phenotypic variance, V(G)/Vp: ratio of genetic variance to phenotypic variance.

^a^ First six principal components.

Supplementary Table 5: Age-, sex-, and population stratification^a^-adjusted bivariate GCTA models for cognition, education, and social class.

|  | n | V(G)_tr1_ | SE | V(G)_tr2_ | SE | C(G)_tr12_ | SE | V(e)_tr1_ | SE | V(e)_tr2_ | SE | C(e)_tr12_ | SE | Vp_tr1_ | SE | Vp_tr2_ | SE | V(G)/Vp_tr1_ | SE | V(G)/Vp_tr2_ | SE | rg | SE |
| --- | --- | --- | --- | --- | --- | --- | --- | --- | --- | --- | --- | --- | --- | --- | --- | --- | --- | --- | --- | --- | --- | --- | --- |
| g : Education | 6,609:6,578 | 0.27 | 0.05 | 0.52 | 0.13 | 0.35 | 0.06 | 0.69 | 0.05 | 1.99 | 0.14 | 0.25 | 0.06 | 0.96 | 0.02 | 2.50 | 0.04 | 0.28 | 0.05 | 0.21 | 0.05 | 0.95 | 0.13 |
| g : SIMD | 6,609:6,533 | 0.28 | 0.05 | 0.17 | 0.05 | 0.06 | 0.04 | 0.67 | 0.05 | 0.81 | 0.05 | 0.18 | 0.04 | 0.96 | 0.02 | 0.98 | 0.02 | 0.29 | 0.05 | 0.18 | 0.05 | 0.26 | 0.16 |
| Education : SIMD | 6,578:6,533 | 0.53 | 0.14 | 0.18 | 0.05 | 0.14 | 0.06 | 1.97 | 0.14 | 0.80 | 0.05 | 0.20 | 0.06 | 2.50 | 0.04 | 0.98 | 0.02 | 0.21 | 0.05 | 0.18 | 0.05 | 0.45 | 0.18 |
| g_f_ : Education | 6,648:6,578 | 0.17 | 0.05 | 0.52 | 0.13 | 0.25 | 0.06 | 0.70 | 0.05 | 1.98 | 0.14 | 0.20 | 0.06 | 0.87 | 0.02 | 2.50 | 0.04 | 0.20 | 0.05 | 0.21 | 0.05 | 0.83 | 0.17 |
| g_f_ : SIMD | 6,648:6,533 | 0.18 | 0.05 | 0.18 | 0.05 | 0.03 | 0.04 | 0.69 | 0.05 | 0.81 | 0.05 | 0.17 | 0.04 | 0.88 | 0.02 | 0.98 | 0.02 | 0.21 | 0.05 | 0.18 | 0.05 | 0.18 | 0.19 |
| MHVS : Education | 6,694:6,578 | 7.78 | 1.11 | 0.51 | 0.13 | 1.82 | 0.30 | 12.76 | 1.08 | 2.00 | 0.14 | 1.21 | 0.29 | 20.55 | 0.36 | 2.51 | 0.04 | 0.38 | 0.05 | 0.20 | 0.05 | 0.92 | 0.11 |
| MHVS : SIMD | 6,694:6,533 | 7.94 | 1.12 | 0.17 | 0.05 | 0.48 | 0.18 | 12.62 | 1.09 | 0.82 | 0.05 | 0.57 | 0.18 | 20.56 | 0.36 | 0.98 | 0.02 | 0.39 | 0.05 | 0.17 | 0.05 | 0.42 | 0.14 |

g: general intelligence derived from principal components analysis, SIMD: Scottish Index of Multiple Deprivation, g_f_: general fluid-type intelligence derived from principal components analysis, MHVS: Mill Hill Vocabulary Scale. V(G)_tr_*_i_*: genetic variance for trait *i*, V(e)_tr_*_i_*: residual variance for trait *i*, Vp_tr_*_i_*: phenotypic variance for trait *i*, V(G)/Vp_tr_*_i_*: ratio of genetic variance to phenotypic variance for trait *i*, C(G)_tr_*_i_*: genetic covariance for trait *i*, C(e)_tr_*_i_*: residual covariance for trait *i*, r_G_: genetic correlation.

^a^ First six principal components.

Supplementary Table 6: Age- and sex- and population stratification^a^-adjusted univariate GCTA models for cognition, education, and social class excluding those with depression.

|  | n | V(G) | SE | V(e) | SE | Vp | SE | V(G)/Vp | SE |
| --- | --- | --- | --- | --- | --- | --- | --- | --- | --- |
| *Individual cognitive tests* |  |  |  |  |  |  |  |  |  |
| DST | 5,903 | 36.320 | 11.773 | 157.939 | 11.900 | 194.259 | 3.582 | 0.187 | 0.060 |
| VFT | 5,916 | 26.950 | 8.238 | 107.010 | 8.298 | 133.960 | 2.469 | 0.201 | 0.061 |
| LM | 5,910 | 9.593 | 3.580 | 49.689 | 3.636 | 59.282 | 1.092 | 0.162 | 0.060 |
| MHVS | 5,878 | 7.942 | 1.258 | 12.284 | 1.224 | 20.226 | 0.376 | 0.393 | 0.061 |
|  |  |  |  |  |  |  |  |  |  |
| *General cognitive ability* |  |  |  |  |  |  |  |  |  |
| g | 5,802 | 0.270 | 0.054 | 0.587 | 0.053 | 0.857 | 0.016 | 0.315 | 0.062 |
| g_f_ | 5,838 | 0.178 | 0.047 | 0.588 | 0.047 | 0.766 | 0.014 | 0.233 | 0.061 |
|  |  |  |  |  |  |  |  |  |  |
| *Environmental variables* |  |  |  |  |  |  |  |  |  |
| Education | 5,795 | 0.445 | 0.151 | 2.014 | 0.153 | 2.459 | 0.046 | 0.181 | 0.061 |
| Education (g_f_ adjusted) | 5,653 | 0.272 | 0.141 | 1.978 | 0.144 | 2.250 | 0.042 | 0.121 | 0.062 |
| Education (MHVS adjusted) | 5,695 | 0.109 | 0.125 | 1.938 | 0.129 | 2.047 | 0.038 | 0.053 | 0.061 |
| SIMD | 5,756 | 0.143 | 0.060 | 0.832 | 0.061 | 0.975 | 0.018 | 0.147 | 0.061 |
| SIMD (g_f_ adjusted) | 5,608 | 0.095 | 0.059 | 0.840 | 0.060 | 0.935 | 0.018 | 0.102 | 0.062 |
| SIMD (MHVS adjusted) | 5,645 | 0.095 | 0.058 | 0.827 | 0.059 | 0.921 | 0.017 | 0.103 | 0.063 |
|  |  |  |  |  |  |  |  |  |  |
| *'Turkheimer' estimates* |  |  |  |  |  |  |  |  |  |
| g (below median SIMD) | 2,683 | 0.150 | 0.121 | 0.792 | 0.122 | 0.942 | 0.026 | 0.159 | 0.128 |
| g (above median SIMD) | 2,889 | 0.228 | 0.089 | 0.494 | 0.088 | 0.722 | 0.019 | 0.315 | 0.122 |

DST: Digit Symbol Test, VFT: Verbal Fluency Test, LM: Logical Memory, MHVS: Mill Hill Vocabulary Scale, g: general intelligence derived from principal components analysis, g_f_: general fluid-type intelligence derived from principal components analysis, SIMD: Scottish Index of Multiple Deprivation. V(G): genetic variance, V(e): residual variance, Vp: phenotypic variance, V(G)/Vp: ratio of genetic variance to phenotypic variance.

^a^ First six principal components.

Supplementary Table 7: Age-, sex- and population stratification^a^-adjusted bivariate GCTA models for cognition, education, and social class excluding those with depression.

|  | n | V(G)_tr1_ | SE | V(G)_tr2_ | SE | C(G)_tr12_ | SE | V(e)_tr1_ | SE | V(e)_tr2_ | SE | C(e)_tr12_ | SE | Vp_tr1_ | SE | Vp_tr2_ | SE | V(G)/Vp_tr1_ | SE | V(G)/Vp_tr2_ | SE | r_G_ | SE |
| --- | --- | --- | --- | --- | --- | --- | --- | --- | --- | --- | --- | --- | --- | --- | --- | --- | --- | --- | --- | --- | --- | --- | --- |
| g : Education | 5802:5795 | 0.26 | 0.05 | 0.43 | 0.15 | 0.33 | 0.07 | 0.60 | 0.05 | 2.03 | 0.15 | 0.21 | 0.07 | 0.86 | 0.02 | 2.46 | 0.05 | 0.30 | 0.06 | 0.18 | 0.06 | 1.00 | 0.17 |
| g : SIMD | 5802:5756 | 0.27 | 0.05 | 0.14 | 0.06 | 0.07 | 0.04 | 0.59 | 0.05 | 0.84 | 0.06 | 0.14 | 0.04 | 0.86 | 0.02 | 0.97 | 0.02 | 0.32 | 0.06 | 0.14 | 0.06 | 0.35 | 0.19 |
| Education : SIMD | 5795:5756 | 0.46 | 0.15 | 0.14 | 0.06 | 0.13 | 0.07 | 2.00 | 0.15 | 0.83 | 0.06 | 0.20 | 0.07 | 2.46 | 0.05 | 0.97 | 0.02 | 0.19 | 0.06 | 0.15 | 0.06 | 0.50 | 0.25 |
| g_f_ : Education | 5838:5795 | 0.17 | 0.05 | 0.44 | 0.15 | 0.23 | 0.06 | 0.60 | 0.05 | 2.02 | 0.15 | 0.17 | 0.06 | 0.77 | 0.01 | 2.46 | 0.05 | 0.22 | 0.06 | 0.18 | 0.06 | 0.84 | 0.20 |
| g_f_ : SIMD | 5838:5756 | 0.18 | 0.05 | 0.14 | 0.06 | 0.05 | 0.04 | 0.59 | 0.05 | 0.83 | 0.06 | 0.12 | 0.04 | 0.77 | 0.01 | 0.97 | 0.02 | 0.23 | 0.06 | 0.14 | 0.06 | 0.32 | 0.23 |
| MHVS : Education | 5878:5795 | 7.79 | 1.25 | 0.44 | 0.15 | 1.82 | 0.33 | 12.43 | 1.22 | 2.02 | 0.15 | 1.10 | 0.33 | 20.22 | 0.38 | 2.47 | 0.05 | 0.39 | 0.06 | 0.18 | 0.06 | 0.98 | 0.15 |
| MHVS : SIMD | 5787:5795 | 7.91 | 1.26 | 0.14 | 0.06 | 0.43 | 0.20 | 12.32 | 1.22 | 0.84 | 0.06 | 0.57 | 0.20 | 20.23 | 0.38 | 0.97 | 0.02 | 0.39 | 0.06 | 0.14 | 0.06 | 0.42 | 0.18 |

g: general intelligence derived from principal components analysis, g_f_: general fluid-type intelligence derived from principal components analysis, SIMD: Scottish Index of Multiple Deprivation, MHVS: Mill Hill Vocabulary Scale.

V(G)_tr_*_i_*: genetic variance for trait *i*, V(e)_tr_*_i_*: residual variance for trait *i*, Vp_tr_*_i_*: phenotypic variance for trait *i*, V(G)/Vp_tr_*_i_*: ratio of genetic variance to phenotypic variance for trait *i*, C(G)_tr_*_i_*: genetic covariance for trait *i*, C(e)_tr_*_i_*: residual covariance for trait *i*, r_G_: genetic correlation.

^a^ First six principal components.
